# Measures of puberty in the Avon Longitudinal Study of Parents and Children (ALSPAC) offspring cohort

**DOI:** 10.12688/wellcomeopenres.19793.2

**Published:** 2024-02-13

**Authors:** Jean Golding, Yasmin Iles-Caven, Kate Northstone, Abigail Fraser, Jon Heron

**Affiliations:** 1 Bristol Medical School, University of Bristol, Bristol, Bristol, BS8 2BN, UK

**Keywords:** ALSPAC, puberty, menarche, sexual development, voice break, pubic hair, SITAR, adolescence

## Abstract

**Background:**

When studying the development of children through the preteen years into adolescence, it is often important to link features of their physical and mental health to the stage of puberty at the time. This is complex since individuals vary substantially in the ages at which they reach different pubertal milestones.

**Methods:**

The Avon Longitudinal Study of Parents and Children (ALSPAC) is an ongoing longitudinal cohort study based in southwest England that recruited over 14000 women in pregnancy, with expected dates of delivery between April 1991 and December 1992. From 1999, information on puberty was collected using a number of different methods : (a) A series of annual questionnaires were administered when the index children were aged between eight and 17 years; these were mainly concerned with the physical changes associated with puberty; (b) identification of the age at peak height growth using the SITAR methodology; and (c) retrospective information from the girls on their age at onset of menstruation (menarche).

**Results:**

The advantages and disadvantages of each method are discussed.

**Conclusions:**

The data are available for analysis by interested researchers.

## Introduction

There is considerable evidence that the average age at onset of puberty has been decreasing over time in many parts of the world ( Bräuner *et al.*, 2020; Queiroga *et al.*, 2020). Adding to concerns resulting from these unexplained secular trends are other changes occurring over time in regard to male biology, with increasing rates of testicular cancer ( Huyghe *et al.*, 2003) and decreasing sperm counts ( Levine *et al.*, 2017; Sengupta *et al.*, 2018). These reports raise the question as to why such trends are occurring. A number of reviews have stated that more high-quality data are required, and that studies should be based on populations rather than individuals referred, for example, for investigation of infertility. Longitudinal cohort studies that start before birth present an excellent opportunity to provide such data and allow for detailed descriptions as to the general pubertal development in a geographic population. 

 In this data note we describe one cohort study that has such data available for research: different types of information have been collected on pubertal development by the Avon Longitudinal Study of Parents and Children (ALSPAC). The aim of this paper is to describe the different measures collected, their advantages and disadvantages. Scientists who wish to access the data are invited to refer to the data availability statement and to find the relevant variable names in this document.

## Methods

### Ethical statement

Ethical approval for the study was obtained from the ALSPAC Ethics and Law Committee (ALEC; IRB00003312) and the Local Research Ethics Committees. All methods were performed in accordance with the relevant guidelines and regulations. Informed consent for the use of data collected via questionnaires and clinics was obtained from participants following the recommendations of the ALSPAC Ethics and Law Committee at the time ( Birmingham, 2018). Detailed information on the ways in which confidentiality of the cohort is maintained may be found on the study website: http://www.bristol.ac.uk/alspac/researchers/research-ethics/.

### The ALSPAC cohort

Pregnant women resident in Avon, UK, with expected dates of delivery between 1st April 1991 and 31st December 1992 inclusive were invited to take part in the study. 20,248 pregnancies were subsequently identified as being eligible; the initial number of pregnancies enrolled was 14,541. Of the initial pregnancies, there was a total of 14,676 foetuses, resulting in 14,062 live births and 13,988 children who were alive at one year of age. After various attempts to bolster the initial sample with eligible cases, the total sample size for analyses using any data collected after the age of seven is 15,447 pregnancies, resulting in 15,658 foetuses. Of these 14,901 children were alive at one year of age ( Boyd *et al.*, 2013; Fraser *et al.*, 2013; Northstone *et al.*, 2019).

Information collected relevant to this data note used self-completion questionnaires https://www.bristol.ac.uk/alspac/researchers/our-data/questionnaires/puberty-questionnaires/ mailed to the main carer of the study child and to the child him/herself. As with the development of all ALSPAC questionnaires, many people, including members of the advisory committees, were involved in ensuring the validity of the questions asked, the design of the questionnaires and ethical aspects of the data collection. The questionnaires were sent out with a number (not a name), and on return a computer programme changed that number to a different number denoting the anonymised identification number. Incomplete and missing data was coded as such. Prior to administration the questionnaires were piloted on volunteers, and comments were taken into account in finalising and printing the questionnaires. Accurate information on height was obtained for the study children who attended clinics where they were measured.

Please note that the study website contains details of all the data that are available through a fully searchable data dictionary and variable search tool: http://www.bristol.ac.uk/alspac/researchers/our-data/.

### The SITAR growth statistics

The use of Super-Imposition by Translation And Rotation (SITAR) growth curve modelling has been used as an instrument for the identification of age at peak height velocity as an estimate of age at puberty ( Cole *et al.*, 2010). Height measures were collected by trained ALSPAC staff in clinics for study children between the ages of seven and 20 years and were used to derive a number of variables of potential use in the determination of the growth spurt related to puberty. These were described by Frysz and her colleagues (2018) as follows: “SITAR is a mixed effects shape-invariant growth curve model, consisting of a mean growth curve along with three transformations (size, tempo and velocity), used to describe how each individual differs from the mean curve. The three SITAR parameters are size, reflecting up/down shift from the mean curve; tempo, reflecting left/right shift (on the age scale) which corresponds to the relative timing of puberty based on aPHV (age at Peak Height Velocity), and velocity reflecting stretching/shrinking of the age scale and hence describing differences in the rate at which individuals pass through puberty.”

The SITAR measurements have been used with a number of longitudinal epidemiological studies including ALSPAC and have shown that peak growth velocity occurred earlier in girls than boys ( Elhakeem *et al.*, 2022). The SITAR variables were associated with subsequent bone health ( Elhakeem *et al.*, 2019) and craniofacial measurements, particularly mandibular size ( Queiroga *et al.*, 2020) but little association with cardiovascular indicators at age 25 ( Maher *et al.*, 2021).

### Annual questionnaires through adolescence

The primary aim of the puberty data collection through repeated questionnaires was to evaluate the effects of exposure to environmental chemicals upon defined growth and developmental markers in children. In particular, the aims as stated in the study proposal were to answer the questions: (i) Do prenatal maternal serum levels of persistent organic compounds predict premature sexual maturation in offspring; and (ii) Are there any birth outcomes (birth defects or developmental disabilities) in this cohort that may reflect prenatal exposure to specific environmental chemicals?

The original proposal stated that the ALSPAC data set offered a unique opportunity to evaluate the impact of prenatal and neonatal chemical exposures upon developing neurological and maturational indices. Many of these chemicals can interfere with normal endocrine and neurotransmitter pathways, e.g., persistent organochlorine chemicals including pesticides and PCBs (polychlorinated biphenyls) which cross the placenta and/or are excreted in breast milk.

To time the development of puberty, an established method uses a series of pictures known as Tanner staging ( Marshall & Tanner, 1969; Marshall & Tanner, 1970). The latter questionnaire was developed for studies in the USA; however, the male version showed circumcised penises which was deemed inappropriate for a British population and so the pictures of the development of the penis were redrawn. Tanner staging assigns a numeric rank to the stage of secondary sexual development. Parental report or self-report of a child’s Tanner staging has been validated elsewhere, e.g., for girls the correlations were 0.82 for self-report and 0.85 for maternal report vs clinical assessment ( Brooks-Gunn *et al.*, 1987; see also Morris & Udry, 1980); for boys the self-reported staging had a kappa coefficient of 0.88 ( Duke *et al.*, 1980).

A set of questions to the carer in the first of the male puberty questionnaires was used to determine the presence of malformations of the male genitalia such as hypospadias and undescended testes (cryptorchidism). Although not necessarily related to puberty, these defects are related to endocrine disrupting chemicals and form a component of the original research question (ii) outlined above.

The design of the questionnaires, all of which were named ‘Growing and Changing’ was such that separate questionnaires were produced for boys and girls (the sexes were as defined at birth). Those for boys had covers with pieces of a jigsaw puzzle, and those for the girls a flower spray ( Puberty questionnaires | Avon Longitudinal Study of Parents and Children | University of Bristol). The colour of the covers varied with the sex and age of the participant.

A total of nine versions of this questionnaire were administered at ages varying from 97 months (eight years, one month) to 204 months (17 years) ( Table 1). With the exception of questionnaire no. 7, which was only sent to those children attending the 15-year clinic (to obtain responses to the puberty questionnaire at the same time as the heights were measured by trained clinic staff), they were posted accompanied by other questionnaires. They were designed so that parents could complete them with or without the cooperation of their study child. The ages at completion of the questionnaires are presented in Table 1.

**Table 1.** Timing of completion of the annual puberty questionnaires.

Quest. no.	Date Completed	Age ^a^ Completed (in years)	Nos. returned	
			Boys	Girls
1.	1999-2001	8.1-8.5	2951	3304
2.	2001-2	9.6-9.8	3360	3657
3.	2002-3	10.7-10.8	3159	3482
4.	2003-4	11.7-11.8	2995	3333
5.	2004-6	13.1-13.2	2875	3200
6.	2006-7	14.6-14.8	2290	2873
7. ^b^	2006-8	15.1-15.7	2295	2566
8.	2007-9	16.0-16.2	1941	2814
9.	2008-10	17.0-17.2	1765	2601

^a^ The ages at which 80–90% of respondents had completed their questionnaire. ^b^ This was only sent to children who attended the 15-year clinic.

There was a difference in the numbers of completed questionnaires returned for the different sexes ( Table 1), as girls were more likely to complete the questionnaire than boys. This difference increased with age. Although the response rate itself was lower than for other questionnaires administered during this time period, the sex-difference in response mirrors that seen more widely across the ALSPAC data collection waves.

### Contents common to boy and girl puberty questionnaires

For obvious reasons, most of the questionnaires were different for boys and girls. However, there were a number of common questions ( Table 2). The parents were asked to measure their child’s current weight and height (and given advice on how to measure height accurately). Answers to each of these measures could be given in metric or imperial (imperial measures were then transformed to metric). For questionnaire no. 7, height and weight were omitted as these measurements were measured at the 15-year clinic by trained ALSPAC staff. The participants who attended were asked to complete and bring their puberty questionnaire with them: they were deposited anonymously in a box on arrival.

**Table 2.** Content of the annual questionnaires.

Data collected	Questionnaire No.								
Content common to both Boy/Girl questionnaires	1	2	3	4	5	6	7	8	9
Height	+	+	+	+	+	+	+ ^a^	+	+
Weight	+	+	+	+	+	+	+ ^a^	+	+
Vigorous activity	+	+	+	+	+	+	+	+	+
Age at completion	+	+	+	+	+	+	+	+	+
Who completed the questionnaire	+	+	+	+	+	+	+	+	+
Contents specific for Boys									
Genital stage	+	+	+	+	+	+	+	+	+
Pubic hair stage	+	+	+	+	+	+	+	+	+
Voice change	-	+	+	+	+	+	+	+	+
Hair in armpits	-	-	+	+	+	+	+	+	+
Born with problem of penis or scrotum	+	-	-	-	-	-	-	-	-
No. testes currently in scrotum	+	-	-	-	-	-	-	-	-
Had surgery on genitals	+	-	-	-	-	-	-	-	-
Contents specific for Girls									
Started menstruation	+	+	+	+	+	+	+	+	+
Age at menarche	+	+	+	+	+	+	+	+	+
Date (mm/yy) 1 ^st^ period	+	+	+	+	+	+	+	+	+
Duration bleeding (days)	+	+	+	+	+	+	+	+	+
Length of cycle	+	+	+	+	+	+	+	+	+
Heavy/prolonged bleed ^b^	+	+	+	+	+	+	+	+	+
Severe cramps ^c^	+	+	+	+	+	+	+ ^b^	+	+
Pelvic pains for most of month ^b^	+	+	+	+	+	+	+	+	+
Taken oral contraceptives	+	+	+	+	+	+	+	+	+
Thyroid problem	+	+	+	+	+	+	+	+	+
Hair growing in armpits	-	-	+	+	+	+	+	+	+
Breast development	+	+	+	+	+	+	+	+	+
Pubic hair development	+	+	+	+	+	+	+	+	+

+ question present - question not present; ^a^ Measured at the 15-year clinic examination; ^b^ Supplementary question concerned whether a doctor had been consulted; ^c^ Question changed from ‘severe cramps’ to ‘pain with period’ and a further question on whether mild, moderate or severe. This change only occurs in Questionnaire no. 7.

For all questionnaires a simple question also asked about physical exercise. The wording for the boys was: ‘In the past month, what was the average number of times that your son participated in vigorous physical activity (such as running, football, swimming, athletics)?’; for the girls it was: ‘In the past month, what was the average number of times that your daughter participated in vigorous physical activity (such as running, dance, gymnastics, netball, swimming, or aerobics)?’. For each, the options were: ‘none’; ‘less than once a week’; 1–3 times a week’; ‘4–6 times a week’, and ‘daily’.

At the end of each questionnaire, the date of completion was given. From this, and the child’s date of birth, the age at completion was calculated in months. A note was made as to whether the questionnaire was completed by the child, a parent, someone else, or a combination of individuals.

### Malformations of the male genitalia

In the boys’ first questionnaire only, was a question designed to ascertain whether the boy had any congenital malformation of the genitalia, particularly focussing on undescended testes and hypospadias. The wording was as follows: ‘Sometimes boys are born with something not quite right with their penis or scrotum. Please read the descriptions below and tick all that apply.’ The options given were: ‘There was nothing wrong’; ‘the testes were not in the scrotum (known as undescended testes)’; the hole in the penis was in the wrong place (known as hypospadias)’; and ‘something else’ which the respondent was asked to describe. Two further questions elicited how many testes the boy had in his scrotum now; and whether he had ever undergone an operation on his penis, testes, or scrotum. The detailed answers are available as text but have not yet been coded.

[Note – there have been two publications from ALSPAC on hypospadias using sources such as the mothers’ descriptions of investigations and treatments in the first three years of the child’s life. ( Hughes *et al.*, 2002; North & Golding, 2000).

### Descriptors of male pubertal development

As shown in Table 2, the stage of development of the boy’s genitals and of his pubic hair were measured on each occasion. These used the same set of pictures and definitions, adapted for ALSPAC and redrawn for this study, using the Tanner definitions ( Marshall & Tanner, 1969; Marshall & Tanner, 1970). The question on change of voice was not asked in the first questionnaire, as it was thought to be too early for this to have occurred. Similarly, collection of information on growth of hair in the armpits was started from the 10-year questionnaire.

### Descriptors of female pubertal development

As shown in Table 2, the stage of development of the girls’ breasts, and details of menstruation were recorded on each occasion. These used the same set of pictures and definitions annually, adapted for ALSPAC with pictures redrawn for this study, using the Tanner definitions. As for the boys, collection of information on growth of hair in the armpits was only begun at the 10-year questionnaire stage. In addition, a question on age at menarche was asked of 5,112 girls attending the ALSPAC clinic at age 12.5 years (var ff2092).

## Results

### The SITAR measurements

Height measurements of 10,236 participants at ages from 5 to 20, each of whom had at least one measurement in each of the three time periods: 5 - <19, 19 - <15 and 15 – 20 years had been used to derive the SITAR calculations by Frysz and colleagues (2018). In all 61,290 height measurements were used to derive the SITAR components by Frysz and colleagues (2018). Table 3 indicates a mean age at peak height velocity to be almost two years later for boys than girls.

**Table 3.** The mean [SD] SITAR measurements for the ALSPAC children (from Frysz *et al*., 2018).

Variable	Variable name	All children	Boys	Girls
aPHV * (years)	cf_apv	12.6 [1.3]	13.6 [0.9]	11.7 [0.8]
Peak Velocity (cm/year)	cf_pv	8.8 [1.5]	10.0 [1.1]	7.7 [0.8]
Velocity (cm/year)	cf_velo	0.0 [0.1]	0.0 [0.1]	0.0 [0.1]
Size (cm)	cf_size	0.0 [0.1]	0.0 [6.5]	0.0 [6.0]
Tempo (years)	cf_tempo	0.0 [0.9]	0.0 [0.9]	0.0 [0.9]
N	5,707	5,707	2,688	3,019

*aPHV age at peak height velocity, SD (standard deviation)

### Results from the annual questionnaires

***Genital malformations in boys*.** Table 4 gives the frequency of early genital structural problems and surgical procedures reported in boys. Note that by age eight as many as 7.0% of boys had less than two testes in their scrotum; but only 0.6% were reported to have hypospadias which compares with 0.64% found in ALSPAC when multiple sources of information were used ( North & Golding, 2000).

**Table 4.** The frequency of genital defects with which the boy was born and/or for which he had had surgery as reported at age eight years.

Genital structural defect	Variable	Proportion of boys (n)
At birth	PUB160	8.0% (231)
Undescended testes	PUB161	2.6% (75)
Hypospadias	PUB162	0.6% (16)
Other	PUB163	4.9% (141)
No. testes in scrotum *	PUB164	None: 3.5% (92) One: 3.5% (91) Two: 93.0% (2413)
Had had surgery	PUB165	5.5% (161)

*At time of completion of first puberty questionnaire

***Developmental phases for boys*.** Serious problems with the male Tanner genital stage data came to light when the data from the first five puberty questionnaires were analysed longitudinally. It was found that 27% of boys less than 10 years old appeared to regress in reported genital stage ( Monteilh *et al.*, 2011). This is in contrast with only 3–5% going backwards for each of male pubic hair stage, female breast stage, and female pubic hair stage. In addition, even after exclusion of males who went backwards in genital stage and males under 10 years of age, the estimated ages at transition into Tanner stages 2 or 3 produced by the modelling process are at least a year earlier than expected. It is therefore strongly recommended that the male Tanner genital stage data (variable PUB850) are not used. In contrast, the stages at which pubic hair appeared is assumed to be reliable and has been used to demonstrate that the ages of pubic hair development are influenced by (a) a genetic variant ( Ong *et al.*, 2009a) and (b) growth in the first eight years ( Monteilh *et al.*, 2011).

Despite the longitudinal trend in genital staging showing errors over time, the general trends of the different stages show the expected variation ( Table 5). It can be seen that the modal stages at the different years of age were: at 8–10 years, >80% of boys were at stage 1; at age 10, 95% were at stages 1 or 2; but at ages 12–13 there was a wider difference in staging with 80% of boys at stages 2, 3 or 4. By age 14, 84% were at stages 4 or 5; and at 15, 90% were at this level of development.

**Table 5.** Frequency of reported Tanner stage of male genital development across the nine time points.

Q	Age (y)	Stage 1	Stage 2	Stage 3	Stage 4	Stage 5	N (= 100%)
1	8	29.2%	39.2%	25.9%	5.3%	0.4%	2791
2	9	61.4%	31.2%	5.3%	0.7%	0.1%	3606
3	10	21.0%	37.4%	31.6%	9.6%	0.4%	2892
4	11	10.5%	30.2%	39.0%	18.6%	1.7%	2611
5	12	3.5%	14.9%	34.7%	37.7%	9.2%	2138
6	14	1.0%	4.7%	17.3%	50.7%	26.4%	2873
7	15	0.5%	2.0%	10.6%	51.2%	35.8%	2566
8	16	0.4%	1.1%	7.0%	36.6%	54.9%	1673
9	17	0.3%	0.7%	3.7%	25.4%	69.9%	1525

Q = questionnaire no.; Y = year; N = no. of individuals with data

The proportion of boys starting to grow pubic hair showed similar variation with 92% of 8-year-olds at stage 1 and 95% of 16–17-year-olds at stages 4 or 5 ( Table 6). There was far less certainty concerning the boy’s change of voice at ages 14 or over, with 10.6% of 17-year-olds being uncertain as to whether this had occurred yet ( Table 7).

**Table 6.** Frequency of reported Tanner stage of male pubic hair development across the nine time points.

Q	Age (y)	Stage 1	Stage 2	Stage 3	Stage 4	Stage 5	N (= 100%)
1	8	92.3%	7.4%	<0.3%	-	-	2791
2	9	80.5%	15.1%	2.8%	0.6%	<0.2%	3606
3	10	69.0%	26.4%	4.1%	0.5%	<0.2%	2880
4	11	40.6%	39.8%	14.5%	4.7%	0.4%	2626
5	12/13	13.0%	23.3%	26.3%	31.1%	6.3%	2422
6	14	0.8%	3.0%	11.3%	56.0%	28.8%	2873
7	15	<0.2%	1.2%	5.5%	49.5%	43.6%	2566
8	16	0.3%	0.5%	3.0%	32.5%	63.7%	1673
9	17	<0.3%	0.3%	0.8%	20.2%	78.5%	1525

Q = questionnaire no.; Y = year; N = no. of individuals with data

**Table 7.** Frequency of reported male change in voice across eight time points.

Q	Age (Y)	No change	Changes occasionally	Totally changed	Unsure	N (=100%)
2	9	94.3%	4.1%	<0.2%	1.5%	3321
3	10	92.8%	5.9%	0.2%	1.2%	3107
4	11	82.4%	14.1%	1.1%	2.4%	2902
5	12	51.7%	31.3%	12.9%	4.1%	2774
6	14	9.1%	34.9%	42.3%	13.6%	2256
7	15	11.2%	24.7%	50.2%	13.9%	2246
8	16	13.2%	15.7%	60.8%	10.3%	1916
9	17	22.1%	9.7%	57.7%	10.6%	1750

Q = questionnaire no.; Y = year of age; N = no. of individuals with data

***Developmental phases in girls*.** In contrast to the boys, the developmental phases for girls (as collected using the annual questionnaires) showed the expected increases in phases over time in breast and pubic hair development and onset of menstruation ( Table 8– Table 10).

**Table 8.** Frequency of reported Tanner stage of female breast development across the nine time points.

Q	Age (y)	Stage 1	Stage 2	Stage 3	Stage 4	Stage 5	unsure	N (= 100%)
1	8	85.5%	13.4%	1.0%	<0.2%	-	-	3186
2	9	61.4%	31.2%	5.3%	0.7%	<0.2%	-	3606
3	10	40.0%	36.7%	19.0%	4.1%	0.2%		3437
4	11	12.7%	33.7%	36.5%	15.0%	2.1%		3260
5	12	1.6%	10.1%	35.3%	39.6%	13.4%		3037
6	14	<0.2%	1.4%	16.9%	55.1%	26.5%	-	2793
7	15	<0.2%	0.4%	10.3%	53.1%	36.1%	-	2482
8	16	<0.2%	0.5%	5.6%	45.5%	48.4%	-	2749
9	17	-	0.4%	3.5%	37.0%	59.2%	-	2571

Q = questionnaire no. ; Y = year; N = no. of individuals with data

**Table 9.** Frequency of reported Tanner stage of female pubic hair development across the nine time points.

Q	Age (y)	Stage 1	Stage 2	Stage 3	Stage 4	Stage 5	N (= 100%)
1	8	94.6%	4.9%	0.5%	-	-	3268
2	9	80.5%	15.1%	2.8%	0.6%	<0.2%	3625
3	10	58.1%	27.3%	10.0%	3.6%	1.0%	3426
4	11	27.8%	30.1%	23.1%	13.9%	5.1%	3286
5	12	4.8%	11.3%	22.3%	39.1%	22.5%	3106
6	14	0.4%	0.5%	8.6%	47.9%	42.6%	2768
7	15	0.2%	0.5%	4.1%	40.5%	54.7%	2457
8	16	0.6%	0.2%	2.5%	30.1%	66.6%	2730
9	17	0.7%	0.2%	1.1%	20.8%	77.3%	2551

Q = questionnaire no. ; Y = year; N = no. of individuals with data

**Table 10.** Frequency of reported female menstruation across eight time points.

Q	Age (Y)	Yes	N (=100%)
2	9	0.5%	3624
3	10	2.2%	3459
4	11	16.2%	3306
5	12	61.9%	3185
6	14	96.1%	2861
7	15	98.2%	2556
8	16	99.4%	2812
9	17	99.7%	2585

Q = questionnaire no.; Y = year of age; N = no. of individuals with data

**Figure 1.**
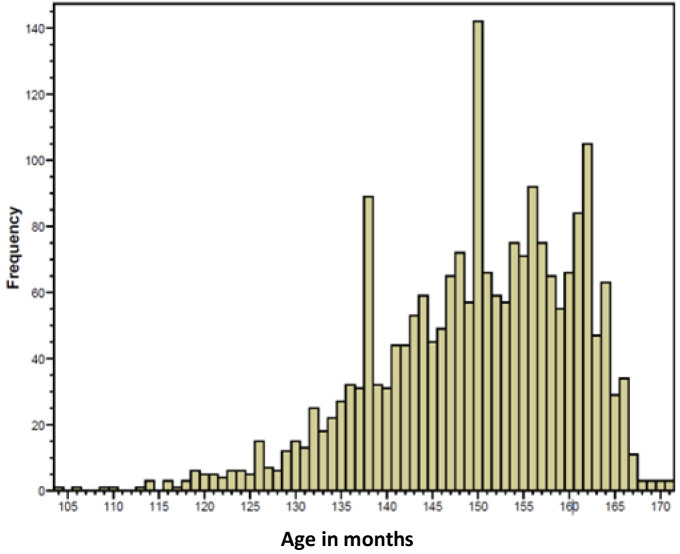
Age (months) at the onset of menstruation ascertained at the 13.5-year clinic.

***Age at menarche*.** As shown in Table 2, the age at which the girl had her first period together with questions concerning details of menstruation were the same in all nine questionnaires. This enabled the age of menarche to be calculated. [However, it should be noted that the age of menarche can also be obtained for the study girls from another source within ALSPAC [questions asked in a clinic visit at age 13.5 years – variable: fg6192 ( Figure 1)]. Some researchers have just used the latter source even though the ages are curtailed ( Ong *et al.*, 2009a and Ong *et al.*, 2009b; Rogers *et al.*, 2010), others have combined the two sources ( Joinson *et al.*, 2011), and the CDC group have just used the longitudinal data from the nine questionnaires (e.g., Adgent *et al.*, 2012; Christensen *et al.*, 2010a; Christensen *et al.*, 2010b; Christensen *et al.*, 2010c; Christensen *et al.*, 2011; Maisonet *et al.*, 2010; Rubin *et al.*, 2009). Combining the two sources is complex, but when Joinson and colleagues (2011) considered the association between menarche and depression they categorised the data from the two data sets into early menarche (<11.5 years), normative (11.5 – 12.5 years) and late onset (>12.5 years).

## Discussion

In this data note we have outlined the data collected on the developmental markers that are available in ALSPAC to estimate the likely onset of puberty for boys and girls. We have indicated that, for boys, the Tanner diagrams of genital development are problematic at early ages, but that age at onset of appearance of pubic hair is probably a valid measure. For girls, the Tanner pictures of breast development and appearance of pubic hair give plausible results, as does the age at menarche, particularly if using the Tanner questionnaires. For both sexes the age at peak height velocity gives ages in close alignment with those of the biological development measures. Correlations between the various measures described here, together with newly derived informative variables can be found for this cohort elsewhere ( Elhakeem *et al*., 2023). Interestingly, the authors also show that the published genetic risk scores for age at menarche are also associated with the different aspects of pubertal development as described here.

### Strengths and limitations

The strength of these data lies in the fact that the population is geographically defined and includes the majority (~80%) of the eligible population. Other strengths include a large sample size, longitudinal design and regular administration of questionnaires on pubertal development (thus reducing the chance of recall bias) with additional availability of data on age at peak height velocity and age at menarche as well as potential confounding factors. Limitations of this study include sample attrition which is strongly associated with socioeconomic disadvantage. An additional drawback is that, at the time of birth, there were very few families of non-white minorities resident in the area (~6%). Consequently, any research results cannot be extrapolated to cover non-white populations in general. As previously stated, there are also problems with the male genital development data. We suggest that researchers collecting male Tanner data by self-completion questionnaires might consider including a subset of participants in which staging is also identified using palpation and observation to address the issue of validity of the self-completion measures used.

This data note provides details of various questionnaire-based measures that were originally designed to be used by researchers investigating possible effects of exposure to endocrine disrupting chemicals. However, they can be applied to any question, concerning the antecedents and long-term consequences of age at onset of puberty in the ALSPAC cohort. In addition, the questions used are recorded here as researchers planning data collection on this topic may find them useful, particularly if planning to compare across cohorts. 

There is probably no one measure that accurately captures ‘age at puberty’, given that puberty is a process, not a binary yes/no event. The various markers described here likely reflect different biological mechanisms to different extents, for example, levels of testosterone, adrenal, growth and gonadal hormones. The availability of multiple measures of pubertal timing may therefore facilitate comparisons that have the potential to yield mechanistic insights.

## Data Availability

ALSPAC data access is through a system of managed open access. The steps below highlight how to apply for access to the data included in this data note and all other ALSPAC data: Please read the ALSPAC access policy ( http://www.bristol.ac.uk/media-library/sites/alspac/documents/researchers/data-access/ALSPAC_Access_Policy.pdf) which describes the process of accessing the data and samples in detail, and outlines the costs associated with doing so. You may also find it useful to browse our fully searchable research proposals database ( https://proposals.epi.bristol.ac.uk/?q=proposalSummaries), which lists all research projects that have been approved since April 2011. Please submit your research proposal ( https://proposals.epi.bristol.ac.uk/) for consideration by the ALSPAC Executive Committee. You will receive a response within 10 working days to advise you whether your proposal has been approved. For data specific to the puberty questionnaires, the variable numbers are given in the following tables. Each variable number should be preceded by PUB. For example, in Table 1, the variable for weight for the 4 ^th^ questionnaire is PUB404. OSF: Measures of puberty in the Avon Longitudinal Study of Parents and Children (ALSPAC) offspring cohort. https://doi.org/10.17605/OSF.IO/3ERKP. ( Golding *et al.*, 2023). This project contains the following extended data: Supplementary Tables.docx. (Variable names for questionnaires). Data are available under the terms of the Creative Commons Attribution 4.0 International license (CC-BY 4.0).
