# Supplementary material for: Measures of puberty in the Avon Longitudinal Study of Parents and Children (ALSPAC) offspring cohort
Source: Wellcome Open Res. 2024 Feb 13;8:453. Originally published 2023 Oct 12. [Version 2] doi: 10.12688/wellcomeopenres.19793.2 (PMC11075130; doi:10.12688/wellcomeopenres.19793.2)
Supplement: Peer review history

# Reviewer report: Reviewer response for version 2

**DOI:** 10.21956/wellcomeopenres.23227.r74383

**Published:** 2024-02-28

**Authors:** Laura Hart

**Affiliations:** 1 Departments of Pediatrics and Medicine, The Ohio State University College of Medicine, Columbus, OH, USA; 2 Center for Child Health Equity and Outcomes Research, The Abigail Wexner Research Institute, Nationwide Children's Hospital, Columbus, OH, USA

I agree with the authors that the edits to the introduction improve the article greatly.

 I understand that some loss of data is to be expected between the children in the cohort who reached one year of age, the children who could be identified for survey completion at 7-8 years of age (and in subsequent rounds), and the number of surveys completed in full.  In order for a reader to confirm the 80% eligibility statement in the discussion, it would be helpful if the authors could provide those numbers in addition to reviewing eligible pregnancies, births, and those reaching one year old, which they have already included.

Are sufficient details of methods and materials provided to allow replication by others?

Yes

Is the rationale for creating the dataset(s) clearly described?

Yes

Are the datasets clearly presented in a useable and accessible format?

Yes

Are the protocols appropriate and is the work technically sound?

Yes

Reviewer Expertise:

My focus is the transition from pediatric to adult health care.

I confirm that I have read this submission and believe that I have an appropriate level of expertise to confirm that it is of an acceptable scientific standard, however I have significant reservations, as outlined above.

# Reviewer report: Reviewer response for version 2

**DOI:** 10.21956/wellcomeopenres.23227.r74384

**Published:** 2024-02-22

**Authors:** Hoi Lun Cheng

**Affiliations:** 1 Academic Department of Adolescent Medicine, The Children's Hospital at Westmead, Westmead, New South Wales, Australia; 2 Faculty of Medicine and Health, Sydney Medical School, Specialty of Child and Adolescent Health, The University of Sydney, Sydney, New South Wales, Australia

Figure 1 histogram - suggest also showing age in years instead of months.

 I think the authors may have missed my final comment in the first review related to the implications of attrition bias. The authors noted that attrition was strongly related to socio-economic status, but there was no further explanation as to how that might affect the data. I provided some potential implications in my last review and I would suggest adding these to the discussion among any others that the authors might be able to come up with.

 Also, I note the following amendment:

*"There is probably no one measure that accurately captures ‘age at puberty’, given that puberty is a process, not a binary yes/no event. The various markers described here likely reflect different biological mechanisms to different extents, for example, levels of testosterone, adrenal, growth and gonadal hormones. The availability of multiple measures of pubertal timing may therefore facilitate comparisons that have the potential to yield mechanistic insights.* *"*

 I feel like this paragraph is not 100% correct. The main purpose of measuring different markers/descriptors is not to show that puberty is not a binary yes/no event, but really to demonstrate that puberty is a broad term that describes maturation of several different hormone systems that manifest as different physical changes (not sure if i've worded this very well but hopefully the authors understand what I'm trying to say).

 Moreover, testosterone is a gonadal hormone, so there is redundancy in the sentence " *levels of testosterone, adrenal, growth and gonadal hormones"*

Are sufficient details of methods and materials provided to allow replication by others?

Yes

Is the rationale for creating the dataset(s) clearly described?

Yes

Are the datasets clearly presented in a useable and accessible format?

Yes

Are the protocols appropriate and is the work technically sound?

Yes

Reviewer Expertise:

Pubertal assessment, adolescent nutrition and obesity

I confirm that I have read this submission and believe that I have an appropriate level of expertise to confirm that it is of an acceptable scientific standard, however I have significant reservations, as outlined above.

# Reviewer report: Reviewer response for version 2

**DOI:** 10.21956/wellcomeopenres.23227.r74385

**Published:** 2024-02-21

**Authors:** Charlotte Wright

**Affiliations:** 1 University of Glasgow, Glasgow, Scotland, UK

I am happy that the authors have considered all the comments and responded to most of them sensibly.

Are sufficient details of methods and materials provided to allow replication by others?

Partly

Is the rationale for creating the dataset(s) clearly described?

Yes

Are the datasets clearly presented in a useable and accessible format?

Yes

Are the protocols appropriate and is the work technically sound?

Yes

Reviewer Expertise:

Assessment of growth and nutritional status, Child nutrition, epidemiology

I confirm that I have read this submission and believe that I have an appropriate level of expertise to confirm that it is of an acceptable scientific standard.

# Reviewer report: Reviewer response for version 1

**DOI:** 10.21956/wellcomeopenres.21924.r69486

**Published:** 2023-11-24

**Authors:** Hoi Lun Cheng

**Affiliations:** 1 Academic Department of Adolescent Medicine, The Children's Hospital at Westmead, Westmead, New South Wales, Australia; 2 Faculty of Medicine and Health, Sydney Medical School, Specialty of Child and Adolescent Health, The University of Sydney, Sydney, New South Wales, Australia

This manuscript details the collection of and available data pertaining to pubertal development in the ALSPAC cohort. It was difficult to appraise using standard metrics relevant to a quantitative study.

 First of all, I feel that this manuscript is important in that ALSPAC is indeed a highly valuable longitudinal adolescent health dataset that undoubtedly has much more to give.

 A clear limitation of the dataset that I feel has not been adequately disclosed, however, is the self-reported nature of Tanner staging. The gold standard method is Tanner staging via palpation by a trained clinician. ALSPAC falls short in this respect, but understandably this is the compromise made for the sample size and longitudinality of the cohort, and also for ethical reasons. However, I'm failing to see this acknowledged adequately as a limitation in the discussion section. This is an important point as readers of this manuscript who may be interested in accessing the ALSPAC data may have expertise in other areas of adolescent health, but not necessarily in the nuances of different pubertal assessment methodologies.

 It would also be useful to relate each pubertal descriptor used in ALSPAC to the underlying hormones responsible for such development. For example, pubertal height growth relates more to the synergistic actions of growth hormone and testosterone, the tanner breast/genital measures are more reflective of gonadal hormones, whereas pubic hair development more to adrenal hormones. Depending on the health outcome of research interest, one descriptor may be more relevant to others. It would be important to point out that any future users of the ALSPAC data carefully consider which pubertal descriptor(s) is most appropriate for their research question, as these descriptors do not measure the same thing and cannot be used interchangeably.

 I commend the authors for being upfront about the quality of the male Tanner stage data. My team had similar experiences with our adolescent males (although to a lesser degree), but the fact that challenges relating to self-reported teen male data was documented in the manuscript is valuable for any future research in this area.

 Finally, I just have a question as authors alluded to attrition related to "socio-economic disadvantage" would it be useful to expand on this further and talk about the implications of this attrition bias? i.e. those with lower SES who dropped out of the study may be the ones experiencing earlier puberty due to potential childhood stressors, higher rates of obesity etc.

Are sufficient details of methods and materials provided to allow replication by others?

Yes

Is the rationale for creating the dataset(s) clearly described?

Yes

Are the datasets clearly presented in a useable and accessible format?

Yes

Are the protocols appropriate and is the work technically sound?

Yes

Reviewer Expertise:

Pubertal assessment, adolescent nutrition and obesity

I confirm that I have read this submission and believe that I have an appropriate level of expertise to confirm that it is of an acceptable scientific standard, however I have significant reservations, as outlined above.

# Response

**Published:** 2024-01-11

**Authors:** Yasmin Iles-Caven

**Affiliations:** University of Bristol, UK

This manuscript details the collection of and available data pertaining to pubertal development in the ALSPAC cohort. It was difficult to appraise using standard metrics relevant to a quantitative study. 

 First of all, I feel that this manuscript is important in that ALSPAC is indeed a highly valuable longitudinal adolescent health dataset that undoubtedly has much more to give.

 A clear limitation of the dataset that I feel has not been adequately disclosed, however, is the self-reported nature of Tanner staging. The gold standard method is Tanner staging via palpation by a trained clinician. ALSPAC falls short in this respect, but understandably this is the compromise made for the sample size and longitudinality of the cohort, and also for ethical reasons. However, I'm failing to see this acknowledged adequately as a limitation in the discussion section. This is an important point as readers of this manuscript who may be interested in accessing the ALSPAC data may have expertise in other areas of adolescent health, but not necessarily in the nuances of different pubertal assessment methodologies. *Many thanks for your important observations. You are right in that, because of the considerable cost of doing so, the study did not include palpation of the male genitalia, although this was discussed at the planning stage. In retrospect it would have been valuable to palpate a random sample of boys and compare the results with the questionnaire data, and we have now mentioned that in the limitation section of the discussion. * It would also be useful to relate each pubertal descriptor used in ALSPAC to the underlying hormones responsible for such development. For example, pubertal height growth relates more to the synergistic actions of growth hormone and testosterone, the tanner breast/genital measures are more reflective of gonadal hormones, whereas pubic hair development more to adrenal hormones. Depending on the health outcome of research interest, one descriptor may be more relevant to others. It would be important to point out that any future users of the ALSPAC data carefully consider which pubertal descriptor(s) is most appropriate for their research question, as these descriptors do not measure the same thing and cannot be used interchangeably. *Thank you very much for pointing this out. We have inserted a paragraph at the end of the Discussion to cover this important point.*

# Reviewer report: Reviewer response for version 1

**DOI:** 10.21956/wellcomeopenres.21924.r69487

**Published:** 2023-11-10

**Authors:** Laura Hart

**Affiliations:** 1 Departments of Pediatrics and Medicine, The Ohio State University College of Medicine, Columbus, OH, USA; 2 Center for Child Health Equity and Outcomes Research, The Abigail Wexner Research Institute, Nationwide Children's Hospital, Columbus, OH, USA

In this report, the authors compare their strategies for determining the stage of puberty for each participant in the ALSPAC cohort.

 The introduction is focused mostly on precocious puberty for the first few sentences, which is not mentioned in the abstract. It becomes evident toward the end of the introduction that the aim is to study puberty longitudinally in the cohort so that the data can inform our understanding of precocious puberty, and so the link comes together eventually. As a reader, I would have appreciated the link being made in both the abstract and the introduction. At the same time, I found the reasoning for the study described in the abstract much more compelling. I wonder if the authors might consider focusing the introduction on the idea that study of adolescent health overall would benefit from understanding findings relative to where an adolescent is in their pubertal development.

 This is in part because the focus on precocious puberty raises a methodologic concern for me, which is that the authors didn't start collecting data until age 8, which is late for a focus on precocious puberty. If the goal is to study precocious puberty, wouldn't data collection have needed to start sooner? Similarly, if the interest is in studying precocious puberty, why wait until 10 years of age to ask about hair in the arm pits?

 In the strengths and limitations, the authors mention that they have data on 80% of the eligible population, but it is not clear to me how they got that number. The initial cohort included 15,000 births and the highest number of returned surveys in Table 1 is ~7,000, which is less than 50%. If they could clarify that point, I would appreciate it.

Are sufficient details of methods and materials provided to allow replication by others?

Yes

Is the rationale for creating the dataset(s) clearly described?

Yes

Are the datasets clearly presented in a useable and accessible format?

Yes

Are the protocols appropriate and is the work technically sound?

Yes

Reviewer Expertise:

My focus is the transition from pediatric to adult health care.

I confirm that I have read this submission and believe that I have an appropriate level of expertise to confirm that it is of an acceptable scientific standard, however I have significant reservations, as outlined above.

# Response

**Published:** 2024-01-11

**Authors:** Yasmin Iles-Caven

**Affiliations:** University of Bristol, UK

In this report, the authors compare their strategies for determining the stage of puberty for each participant in the ALSPAC cohort.

 The introduction is focused mostly on precocious puberty for the first few sentences, which is not mentioned in the abstract. It becomes evident toward the end of the introduction that the aim is to study puberty longitudinally in the cohort so that the data can inform our understanding of precocious puberty, and so the link comes together eventually. As a reader, I would have appreciated the link being made in both the abstract and the introduction. At the same time, I found the reasoning for the study described in the abstract much more compelling. I wonder if the authors might consider focusing the introduction on the idea that study of adolescent health overall would benefit from understanding findings relative to where an adolescent is in their pubertal development.  This is in part because the focus on precocious puberty raises a methodologic concern for me, which is that the authors didn't start collecting data until age 8, which is late for a focus on precocious puberty. If the goal is to study precocious puberty, wouldn't data collection have needed to start sooner? Similarly, if the interest is in studying precocious puberty, why wait until 10 years of age to ask about hair in the arm pits? *We are grateful to the Reviewer for raising this issue. She is correct in thinking that although the initiators of the pubertal data collection had intended to look at precocious puberty, bureaucratic delays meant that the children were already 8 years of age before we could start administering the questionnaires. We have therefore omitted any mention of precocious puberty in the text, and feel that the reasons behind the study now develop more rationally.* In the strengths and limitations, the authors mention that they have data on 80% of the eligible population, but it is not clear to me how they got that number. The initial cohort included 15,000 births and the highest number of returned surveys in Table 1 is ~7,000, which is less than 50%. If they could clarify that point, I would appreciate it. *Apologies for the confusion. The statement was intended to refer to the enrolment rate within the population at risk. As will be appreciated, when identifying eligible pregnancies as opposed to births, the numbers are approximate and vary according to gestation, pregnancy outcome and the time frame. In general, excluding the poor responses at the start and end of the enrolment period, 80% appears to be a reasonable estimate of the number of viable pregnancies enrolled* *.*

# Reviewer report: Reviewer response for version 1

**DOI:** 10.21956/wellcomeopenres.21924.r68625

**Published:** 2023-10-25

**Authors:** Charlotte Wright

**Affiliations:** 1 University of Glasgow, Glasgow, Scotland, UK

This interesting article describes the various ways in which puberty was assessed in the ALSPAC longitudinal cohort and gives a clear overall account of the data gathered and what it showed. The articles states an aim to consider the advantages and disadvantages of different assessment approaches, but apart from a very interesting account of the difficulties of assessing genital size, it does not yet really compare the different approaches.

 Areas that need more comparison are:

1. Age at menarche: They describe two approaches to recording this, one being prospective self-completion questions annually to age 17 (table 10) and the other being a retrospective question at age 13.5 (figure 1) which would be bound to miss girls reaching menarche going beyond that age. While the results in table 10 suggest that the latter approach might miss only around 4% of children, this is not stared explicitly. Instead of showing figure 1 it would be better to compare the results in one table and also show the result of the combined method mentioned in the text.
2. Pubertal changes in boys: Given the challenges in assessing penile size, it would be interesting to compare these to the data on pubic hair, voice breaking and underarm hair – for which no data are shown currently. Could these be combined in a figure and could their inter-correlation be assessed in some way – e.g looking at the correlation of age at which boys entered stage 2 or had any voice change.

The use of the SITAR method is novel and interesting, but the results need a fuller explanation. A limitation of assessing peak height velocity is the availability of height data at the right ages. We need to be shown the numbers of heights at each age and the % of the children included with heights at most or all ages.

 In the design of the new UK charts a simplified pubertal assessment approach was adopted with just 2 stages for each gender – ***starting*** puberty, which constituted any pubic hair on either gender or penile enlargement in boys or development of breast in girls, and ***completion*** of puberty being menarche in girls or voice breaking or underarm hair in boys. I am aware that this has not been fully written up yet, but as well as the brief mention cited here ^1^, I understand that it has been described in both Oxford Textbook of Medicine and Oxford Textbook of Endocrinology and Encyclopedia of Endocrine Diseases in chapters authored by Professor Gary Butler. It looks to me as if this data could be used to describe the age of each stage in the same way – and I wonder if this new approach should be mentioned here at least.

 Having added or changed the above elements I would like to see either text or a table that makes recommendations about which questions to include for what purpose. 

 The current section on strengths and the limitations discuss the ALSPAC *data* as a resource, yet the data are now quite old and already much used. Surely the main point is to help people in their planning of future studies. In that case it is the strengths and limitations of the *questions* used that matter.

 More minor points:

1. Ages given in months in table 1 would be much clearer if used decimal age in years.
2. Table 3 has a mistake in bottom line – values for total N and boys have been displaced sideways.
3. I was unconvinced by the relevance in a paper on puberty assessment of including information on genital defects or activity levels.

Are sufficient details of methods and materials provided to allow replication by others?

Partly

Is the rationale for creating the dataset(s) clearly described?

Yes

Are the datasets clearly presented in a useable and accessible format?

Yes

Are the protocols appropriate and is the work technically sound?

Yes

Reviewer Expertise:

Assessment of growth and nutritional status, Child nutrition, epidemiology

I confirm that I have read this submission and believe that I have an appropriate level of expertise to confirm that it is of an acceptable scientific standard, however I have significant reservations, as outlined above.

## References

[1] : Using the new UK-WHO growth charts. *Paediatrics and Child Health* .2014;24(3) : 10.1016/j.paed.2013.09.012 97-102 10.1016/j.paed.2013.09.012

# Response

**Published:** 2024-01-11

**Authors:** Yasmin Iles-Caven

**Affiliations:** University of Bristol, UK

This interesting article describes the various ways in which puberty was assessed in the ALSPAC longitudinal cohort and gives a clear overall account of the data gathered and what it showed. The articles states an aim to consider the advantages and disadvantages of different assessment approaches, but apart from a very interesting account of the difficulties of assessing genital size, it does not yet really compare the different approaches.  Areas that need more comparison are:

1. Age at menarche: They describe two approaches to recording this, one being prospective self-completion questions annually to age 17 (table 10) and the other being a retrospective question at age 13.5 (figure 1) which would be bound to miss girls reaching menarche going beyond that age. While the results in table 10 suggest that the latter approach might miss only around 4% of children, this is not stated explicitly. Instead of showing figure 1 it would be better to compare the results in one table and also show the result of the combined method mentioned in the text.

*Thank you for raising this issue. Although there is no clear way of combining these two data sets,  we have now described in the text the way  in which another author has addressed the problem  (see end of Results section).*

1. Pubertal changes in boys: Given the challenges in assessing penile size, it would be interesting to compare these to the data on pubic hair, voice breaking and underarm hair – for which no data are shown currently. Could these be combined in a figure and could their inter-correlation be assessed in some way – e.g looking at the correlation of age at which boys entered stage 2 or had any voice change.

*The inter-correlations between the various ALSPAC measures of puberty have been explored in detail by Elhakeem and colleagues (2023). Although available on line we did not wish to quote before they have been published after peer review. Reference to this is now included in the first paragraph of the Discussion. Their results also show, interestingly, that the genetic risk score for age at menarche is positively associated with the age at onset of the other  puberty measures, including those of the boys as well as of the girls. * The use of the SITAR method is novel and interesting, but the results need a fuller explanation. A limitation of assessing peak height velocity is the availability of height data at the right ages. We need to be shown the numbers of heights at each age and the % of the children included with heights at most or all ages. *We have expanded the information in the paper to say: ‘* *Height measurements of 10,236 participants at ages from 5 to 20, who had at least one measurement in each of the three time periods: 5 - <19, 19 - <15 and 15 – 20 years,* *had been used to derive the SITAR calculations by Frysz and colleagues (2018). In all,* *61,290 height measurements were used.’* **In the design of the new UK charts a simplified pubertal assessment approach was adopted with just 2 stages for each gender – ***starting*** puberty, which constituted any pubic hair on either gender or penile enlargement in boys or development of breast in girls, and ***completion*** of puberty being menarche in girls or voice breaking or underarm hair in boys. I am aware that this has not been fully written up yet, but as well as the brief mention cited here ^1^ , I understand that it has been described in both Oxford Textbook of Medicine and Oxford Textbook of Endocrinology and Encyclopedia of Endocrine Diseases in chapters authored by Professor Gary Butler. It looks to me as if this data could be used to describe the age of each stage in the same way – and I wonder if this new approach should be mentioned here at least. *Thank you for telling us about this. On reflection, this Data Note is for an international audience and we feel that reference to UK standards might seem somewhat parochial if their justification has not yet been published in a peer review journal* *.* Having added or changed the above elements I would like to see either text or a table that makes recommendations about which questions to include for what purpose. *Thank you for this suggestion. We have tried to address the question towards the end of the Discussion.*

 The current section on strengths and the limitations discuss the ALSPAC *data* as a resource, yet the data are now quite old and already much used. Surely the main point is to help people in their planning of future studies. In that case it is the strengths and limitations of the *questions* used that matter. *Conversely we feel that puberty being only one feature in the lifecourse means that the value of these data in the future will be in identifying ways in which the timing of puberty of an individual may have a profound effect on their subsequent physical and mental health. * More minor points: Ages given in months in table 1 would be much clearer if used decimal age in years. *This has now been done* Table 3 has a mistake in bottom line – values for total N and boys have been displaced sideways. *Thank you for spotting this. It has now been corrected* *.*

*I was unconvinced by the relevance in a paper on puberty assessment of including information on genital defects or activity levels.* *Apart from the importance of collecting information on genital defects in order to determine links with endocrine disruptors, information on genital defects and operations on genitalia was collected since researchers may wish to exclude boys with such defects from analyses on pubertal state. The details on exercise/activity were included in the puberty questionnaires since it may be that researchers may wish to determine whether the degree of such activities may delay (or advance) the onset of different stages of puberty. We have described these variables here so that researchers looking at the relationships between exercise and body mass index on different aspects of the onset of puberty will be aware that the data were collected at the same time points as the Tanner descriptions.* **Are sufficient details of methods and materials provided to allow replication by others?  **Partly *We hope that our answers to your queries and comments will now assure you that the information in this Data Note will allow replication by others.*
